# Association Between Epstein–Barr Virus Infection and PD-L1 Expression in Gastric Cancer: Prevalence, Clinicopathological Features, and Prognostic Implications

**DOI:** 10.3390/cancers17091492

**Published:** 2025-04-29

**Authors:** Jirapat Wonglhow, Jarukit Tantipisit, Panu Wetwittayakhlang, Patrapim Sunpaweravong, Chirawadee Sathitruangsak, Kanet Kanjanapradit, Phatcharaporn Thongwatchara, Arunee Dechaphunkul

**Affiliations:** 1 Division of Medical Oncology, Department of Internal Medicine, Faculty of Medicine, Prince of Songkla University, Songkhla 90110, Thailand; jirapat.jw@gmail.com (J.W.); spatrapi@medicine.psu.ac.th (P.S.); sjirawadee@gmail.com (C.S.); thongwatchara.p@hotmail.com (P.T.); 2 Department of Pathology, Faculty of Medicine, Prince of Songkla University, Songkhla 90110, Thailand; medew.jarukit@gmail.com (J.T.); kankanet99@hotmail.com (K.K.); 3 Division of Gastroenterology and Hepatology, Department of Internal Medicine, Faculty of Medicine, Prince of Songkla University, Songkhla 90110, Thailand; wet.panu@gmail.com

**Keywords:** Epstein–Barr virus, PD-L1 expression, biomarker, gastric cancer, prognosis, survival outcome

## Abstract

Background: Epstein–Barr virus-associated gastric cancer (EBVaGC) represents a distinct molecular subgroup with potential responsiveness to immunotherapy approved for programmed death-ligand 1 (PD-L1)-positive gastric cancer. This retrospective study aimed to assess the prevalence and association between EBVaGC and PD-L1 positivity among patients with gastric adenocarcinoma treated at a university hospital in Southern Thailand from January 2017 to October 2023. Methods: The EBV status of the patients and PD-L1 expression were determined using in situ hybridization and immunohistochemistry, respectively. Results: The prevalence of EBVaGC was 4.5% among 132 patients, whereas 9.1% of patients exhibited a PD-L1 combined positive score (CPS) of ≥1, with no significant association observed between them. EBVaGC was more prevalent in males, non-antral tumors, diffuse/mixed histologic subtypes, and poorly differentiated tumors. Median overall survival for patients with EBVaGC and PD-L1 CPS ≥ 1 was 9.48 and 14.19 months, respectively, compared with 10.32 and 9.79 months for those with non-EBVaGC (hazard ratio: 1.24; 95% CI: 0.50–3.04; *p* = 0.645) and PD-L1 CPS < 1 (hazard ratio: 0.82; 95% CI: 0.40–1.69; *p* = 0.590), respectively. Conclusions: Our findings revealed a low prevalence of EBVaGC and PD-L1 positivity in Thailand, with no significant association or survival impact observed. These findings highlight the regional variation in these biomarkers and support EBV as an independent biomarker from PD-L1. However, further research, particularly studies evaluating immunotherapy outcomes, is warranted to clarify the predictive and clinical significance of EBV in gastric cancer.

## Simple Summary

This study investigates the prevalence and relationship between Epstein–Barr virus-associated gastric cancer (EBVaGC) and PD-L1 expression in patients with gastric cancer in Thailand. EBVaGC is a distinct molecular subtype with potential responsiveness to immunotherapy. However, little is known about its prevalence and prognostic role in different regions and ethnicities. The findings revealed a low prevalence of both EBVaGC (4.5%) and PD-L1 positivity (9.1%), with no significant association or survival impact. This study underscores regional variation in these biomarker expressions and highlights the need for further research to clarify their prognostic and therapeutic value, particularly in the context of immunotherapy. These insights may inform personalized treatment strategies and improve outcomes in gastric cancer management globally.

## 1. Introduction

Gastric cancer is the fifth most commonly diagnosed malignancy and the fourth leading cause of cancer-related mortality worldwide as of 2020 [1]. Recent clinical advancements by The Cancer Genome Atlas (TCGA) introduced a comprehensive molecular classification system for gastric cancer, identifying four distinct subtypes based on extensive gene expression profiling. These subtypes include Epstein–Barr virus (EBV)-positive tumors, microsatellite instability (MSI) tumors, genomically stable tumors, and chromosomally unstable tumors [2], thereby enhancing the understanding of the heterogeneity of gastric cancer. Moreover, this classification may improve clinical outcomes by aiding in the identification of potential prognostic markers and enabling more personalized therapeutic strategies, particularly for EBV-positive and MSI subtypes [3].

EBV-associated gastric cancer (EBVaGC) is characterized by several distinct features, including lymphocytic infiltration, programmed death-ligand 1 (PD-L1) expression, DNA hypermethylation, and *PIK3CA* mutations [2]. Consequently, patients with EBVaGC often exhibit elevated PD-L1 expression [4,5,6,7,8,9], suggesting potentially favorable responsiveness to immune checkpoint inhibitors [10,11]. Therefore, EBV positivity may serve as a criterion for selecting patients for immunotherapy-based treatments, for which further studies are warranted.

PD-L1 expression is more frequently observed in certain gastric cancer subgroups, including HER2-positive, EBV-associated, and MSI tumors [12]. Subsequently, PD-L1 is a potentially reliable predictor of response to anti-PD-1 or PD-L1 immunotherapy in various cancers, including gastric cancer [13,14]. A combination of immunotherapy and chemotherapy, with or without targeted therapy, has been approved as a first-line treatment for metastatic HER2-negative [15,16] or HER2-positive gastric adenocarcinoma [17]. Nevertheless, the prognostic significance of PD-L1 expression in gastric cancer remains uncertain, despite the existence of significant research [18].

EBV infection and PD-L1 expression are individually associated with gastric cancer; however, their inter-relationship remains controversial, with conflicting evidence likely attributable to limited data and regional variations. Therefore, this study aimed to investigate the prevalence and association of EBVaGC and PD-L1 expression in gastric cancer in Thailand, along with their clinicopathological features and prognostic implications.

## 2. Materials and Methods

### 2.1. Study Participants

We retrospectively reviewed the medical records of patients with gastric cancer diagnosed between January 2017 and October 2023 at Songklanagarind Hospital. Data were retrieved from the pathological archives of the Department of Pathology, Faculty of Medicine, Prince of Songkla University. The main inclusion criteria were as follows: (i) histologically confirmed adenocarcinoma or poorly differentiated carcinoma of the stomach or esophagogastric junction, and (ii) availability of formalin-fixed paraffin-embedded tissue (FFPE) blocks for analysis at the Department of Pathology, Faculty of Medicine, Prince of Songkla University. Patients without sufficient tissue samples for the further testing of EBV-encoded small RNA (EBER) and PD-L1 expression and those with missing clinical and tumor characteristic information in the hospital information system of Songklanagarind Hospital were excluded.

Patient information, including baseline clinical characteristics such as age at diagnosis, sex, body mass index (BMI), Eastern Cooperative Oncology Group (ECOG) performance status, smoking and alcohol use, comorbid conditions, clinical presentation, and primary tumor location, was retrieved from electronic medical records via the hospital information system at Songklanagarind Hospital. Additional data encompassing tumor, node, and metastasis staging (group staging defined by the 8th edition of the American Joint Committee on Cancer), histopathological details such as tumor differentiation, Lauren classification, HER2 status, lymphovascular and perineural invasion, and cancer treatments, including surgery, radiotherapy, and systemic therapies, were also obtained.

### 2.2. Procedures

#### 2.2.1. Sample Processing

All hematoxylin and eosin-stained slides were reviewed, and representative tumor tissue samples were selected for each case. The tissue microarray (TMA) method was employed to process samples from surgical specimens, whereas biopsy specimen tissues from FFPE blocks were cut into 3 μm thick sections. For TMA, corresponding areas were punched out from individual FFPE tumor blocks and arranged using a Quick Ray manual TMA (Unitma, Seoul, Korea) with a 2 mm needle. Finally, the TMA blocks were sliced into 3 μm thick sections.

Tissue histopathological samples from each patient were subjected to EBER in situ hybridization (ISH) and PD-L1 expression analysis using immunohistochemical (IHC) staining with 22C3 at the Department of Pathology, Faculty of Medicine, Prince of Songkla University.

The EBER ISH and PD-L1 expression analysis results were independently reviewed by two pathologists. In cases of disagreement, the slides were reanalyzed by both investigators using a multiheaded microscope until a consensus was reached.

#### 2.2.2. EBER ISH

EBV status was determined via ISH with probes against EBER (PB0589, Leica Biosystems, Melbourne, Australia), a highly sensitive method for detecting latent EBV infection in FFPE tissue samples, using the automated BOND-MAX system following the manufacturer’s instructions. Briefly, 3 μm FFPE tissue sections were deparaffinized using BOND dewax solution, pretreated for target retrieval using BOND enzyme, and hybridized with EBER probes. Signal detection was performed using an anti-fluorescein isthiocyanate antibody and horseradish peroxidase polymer-conjugated anti-rabbit antibody, followed by chromogenic substrate (3,3′-Diaminobenzidine tetrahydrochloride) development. Both EBV-positive and EBV-negative control tissues were included in each run to ensure assay validity. A sample was considered EBV-positive if tumor cell nuclei demonstrated distinct dark brown chromogenic staining, while the absence of nuclear staining in tumor cells was interpreted as EBV negativity.

#### 2.2.3. PD-L1 IHC

The primary antibody used for PD-L1 detection was clone 22C3 (DAKO, Carpinteria, CA, USA). IHC staining was conducted following the manufacturer’s technical manual. Specimens were evaluated based on the percentage of stained tumor cells (TCs) and tumor-infiltrating immune cells (TIICs). Both negative and positive in-house controls were included for each slide. Any membranous staining of TCs and any membranous and/or cytoplasmic staining of TIICs were considered positive. Necrotic and apoptotic TCs, as well as plasma cells, neutrophils, and fibroblasts, were excluded from evaluation. PD-L1 expression was assessed based on the combined positive score (CPS). A CPS of ≥1 was considered positive, with additional evaluations at cutoff points of 5 and 10 [15,16].

### 2.3. Outcomes

The primary objective of this study was to determine the prevalence of EBV and its association with PD-L1 expression in patients with gastric cancer. Secondary objectives included the assessment of clinicopathological features and prognostic implications, including real-world survival outcomes. Overall survival (OS) was defined as the duration from the date of confirmed histopathological diagnosis until death from any cause.

### 2.4. Statistical Analysis

For baseline characteristics, continuous variables are expressed as median with interquartile range or mean with standard deviation, depending on data distribution. Categorical variables are expressed as frequencies and percentages. Fisher’s exact test was used to assess the association between EBV infection and PD-L1 expression. Furthermore, we evaluated the clinicopathological features of EBVaGC and PD-L1-positive gastric cancer, and prognostic factors for OS were identified using univariate and multivariate Cox regression models. Survival curves were generated using the Kaplan–Meier method and compared using the log-rank test. All statistical analyses were performed using R version 4.3.1 (R Foundation, Vienna, Austria). Two-sided *p*-values were calculated, and a *p*-value of <0.05 was considered statistically significant.

## 3. Results

### 3.1. Patient Characteristics

A total of 132 patients were included in this study, comprising 56 with localized-stage disease, 72 with metastatic-stage disease, and 4 with unknown stage. Patient and tumor characteristics are summarized in Table 1. The median age was 63.2 years, with a predominance of male patients. Tumor locations were evenly distributed across the proximal, middle, and distal stomach, whereas 7.5% had diffuse involvement. Most tumors were poorly differentiated, with signet ring cell features in approximately 25% of cases. Based on the Lauren classification, half of the patients exhibited the diffuse subtype, followed by mixed and intestinal subtypes. *Helicobacter pylori* testing was performed in only half of the patients, of whom 30.6% tested positive. HER2 IHC testing was conducted in 29.2% of patients with metastatic disease, all of whom tested negative. Details regarding the number and sites of metastases in patients with metastatic-stage disease are shown in Table S1. Treatment data for localized and metastatic disease are presented in Tables S2 and S3, respectively.

### 3.2. EBV Prevalence and PD-L1 Expression and Their Clinicopathological Features

The prevalence of EBVaGC was 4.5%, comprising 3.5% of localized-stage and 5.6% of metastatic-stage cases. The median age of patients with EBVaGC was 70.6 years, and all were male with a history of smoking. Furthermore, tumors were predominantly located in the middle or proximal stomach, were poorly differentiated, and lacked signet ring cell features. However, there were no statistically significant differences in the clinical or pathological characteristics between patients with EBVaGC and non-EBVaGC (Table 2).

Positive PD-L1 expression (CPS ≥ 1) was observed in 9.1% of cases, including 12.5% and 6.9% in the localized and metastatic stages, respectively. Similarly to EBV status, no statistically significant differences in clinicopathological features were observed between PD-L1-positive and PD-L1-negative subgroups (Table 2).

### 3.3. Association Between EBV Status and PD-L1 Expression in Gastric Cancer

Patients with EBV-positive gastric cancer showed a trend toward increased PD-L1 expression (CPS ≥ 1 and CPS ≥ 5) across all subgroups. However, no statistically significant association was observed between EBV status and PD-L1 expression, either in the overall cohort or within the localized-stage and metastatic-stage subgroups (Table 3).

### 3.4. Survival Outcomes

**Figure 1:**
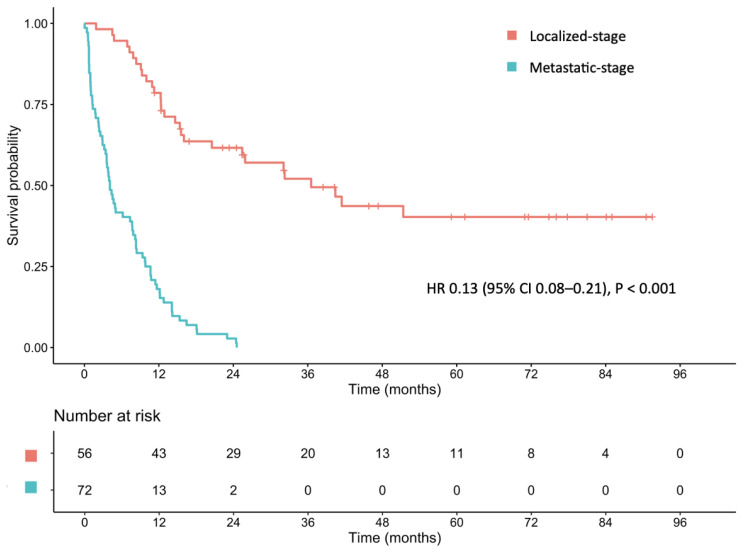
Median overall survival of patients with localized- and metastatic-stage gastric cancer.

The median follow-up time for the entire cohort was 10.32 months, with 30.46 and 9.24 months for the localized-stage and metastatic-stage cohorts, respectively. The median OS was 10.3 months (95% CI: 8.18–12.5) for the entire cohort. The median OS for the localized-stage cohort was 36.53 months (95% CI: 20.53–NA), whereas it was 4.11 months (95% CI: 3.42–7.72) for the metastatic-stage cohort (hazard ratio [HR]: 0.13; 95% CI: 0.08–0.21; *p* < 0.001; Figure 1).

**Figure 2:**
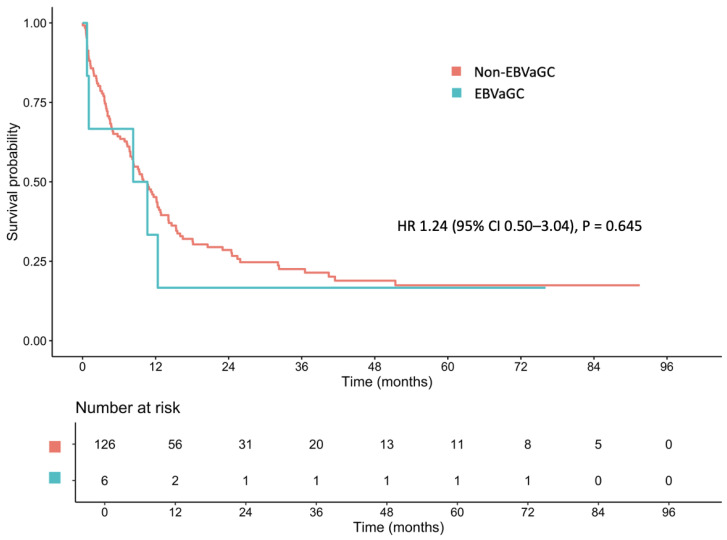
Median overall survival between patients with Epstein–Barr virus-associated gastric cancer (EBVaGC) and non-EBVaGC.

Regarding EBV status, the median OS of patients with EBVaGC and non-EBVaGC was 9.48 months (95% CI: 1.02–NA) and 10.32 months (95% CI: 7.85–12.8), respectively (HR: 1.24; 95% CI: 0.50–3.04; *p* = 0.645; Figure 2). Furthermore, no significant difference in median OS was observed between patients with EBVaGC and non-EBVaGC in the localized-stage cohort (12.35 vs. 36.53 months; HR: 0.85; 95% CI: 0.11–6.23; *p* = 0.869; Figure S1) or the metastatic-stage cohort (4.66 vs. 4.11 months; HR: 0.93; 95% CI: 0.46–3.21; *p* = 0.786; Figure S1).

**Figure 3:**
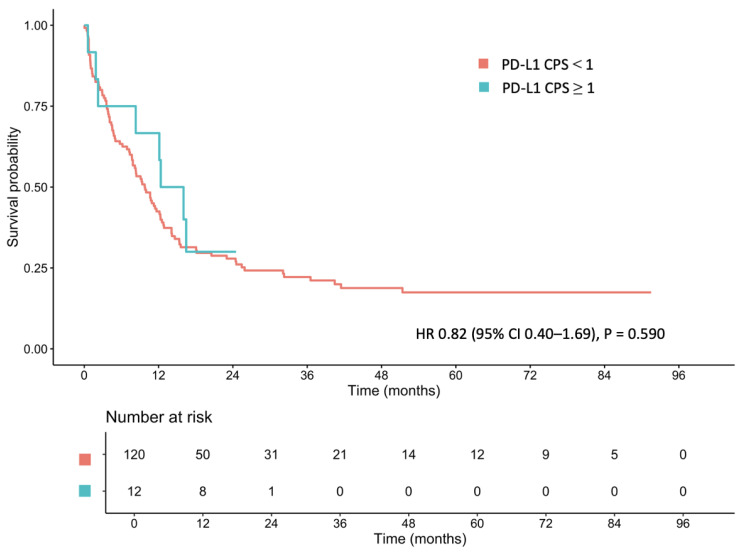
Median overall survival between patients with PD-L1 CPS ≥ 1 and <1 gastric cancer.

With respect to PD-L1 expression, the median OS of patients with PD-L1 CPS ≥ 1 and <1 was 14.19 months (95% CI: 8.31–NA) and 9.79 months (95% CI: 7.85–12.30), respectively (HR: 0.82; 95% CI: 0.40–1.69; *p* = 0.590; Figure 3). Furthermore, no significant difference in median OS was observed between patients with PD-L1 CPS ≥ 1 and <1 in the localized-stage cohort (NA vs. 40.44 months; HR: 0.85; 95% CI: 0.11–6.23; *p* = 0.869) or the metastatic-stage cohort (8.31 vs. 4.11 months; HR: 0.88; 95% CI: 0.46–2.56; *p* = 0.768; Figure S2).

In the metastatic-stage cohort, the median OS significantly increased in patients who received palliative chemotherapy compared with that in patients who received the best supportive care (10.64 vs. 1.31 months; HR 0.11; 95% CI: 0.05–0.20; *p* < 0.001; Figure S3). Furthermore, patients who underwent palliative gastrectomy had improved OS compared to those who did not (12.78 months vs. 3.81 months; HR 0.43; 95% CI 0.21–0.84; *p* = 0.014; Figure S4).

In the localized-stage cohort, the median OS was 40.4 in patients who underwent gastrectomy with adjuvant chemotherapy compared with 51.4 months in those who underwent gastrectomy without adjuvant chemotherapy (HR 0.82; 95% CI 0.33–2.01; *p* = 0.665; Figure S5). Additionally, patients who did not undergo curative surgery but received palliative chemotherapy had a median OS of 12.3 months (HR 4.37; 95% CI 1.21–15.81; *p* = 0.024; Figure S5).

### 3.5. Prognostic Implications

Univariate and multivariate Cox proportional hazard analyses were performed to identify prognostic variables for OS in patients with gastric cancer (Table 4). Neither EBV status nor PD-L1 positivity (CPS ≥ 1) was statistically significant in determining prognosis. In contrast, good ECOG PS (0–1) and localized disease stage were significantly associated with favorable prognosis, whereas hypoalbuminemia was significantly associated with poor prognosis.

## 4. Discussion

This study revealed a 4.5% prevalence of EBVaGC, including 3.5% and 5.6% among patients with localized and metastatic diseases, respectively. Furthermore, PD-L1 positivity (CPS ≥1) was observed in 9.1% of all patients, with 12.5% and 6.9% in those with localized and metastatic diseases, respectively. No significant association between EBV status and PD-L1 expression was identified across different cutoff values or disease stages. No significant difference in median OS was observed between patients with EBVaGC and non-EBVaGC, as well as between those with PD-L1-positive and PD-L1-negative tumors at any disease stage.

The prevalence of EBVaGC has been increasingly documented since the introduction of the TCGA classification of gastric cancer molecular subtypes, which identified EBV as a potential oncogenic driver [2,19]. However, a consensus regarding its role in guiding treatment is lacking, although immunotherapy has been proposed as a potential therapeutic option for this subtype [20,21,22,23,24]. Moreover, information on EBVaGC remains limited, and its prevalence varies significantly worldwide, with higher rates in Western countries (15–30%) compared with Asian regions (5–10%) [4,5,7,8,9,10,25,26,27]. The results of this study conducted in Thailand are consistent with reports from other Asian countries, including Korea, Japan, and China [8,9,10,27], although a slightly lower EBVaGC prevalence (4.5%) was observed. These findings highlight the regional variations in EBVaGC prevalence, suggesting its lower frequency in Asian populations compared with Western populations [20,28].

Although EBVaGC in our study was more frequently observed in male patients, tumors involving non-antral tumor locations, and histologically classified as poorly differentiated with diffuse or mixed subtypes (Lauren classification), these trends did not reach statistical significance. This lack of significance may reflect the limited number of EBVaGC cases in our cohort, reducing statistical power. Nonetheless, the observed trends are consistent with the previously published literature [10,28,29,30].

The prognostic relevance of EBV infection in gastric cancer remains unclear. While some studies report favorable [31,32] or unfavorable [33,34] prognoses associated with EVCaGC, others have found no significant survival differences [35,36]. A meta-analysis demonstrated better survival in EBVaGC compared with non-EBVaGC but reported substantial heterogeneity, likely attributable to regional variations [37]. In this study, median OS did not significantly differ between EBVaGC and non-EBVaGC (9.48 vs. 10.32 months), suggesting that EBV status may not be a reliable prognostic factor.

Currently, immunotherapy has demonstrated clinical benefits in patients with PD-L1-positive gastric cancer [15,16,17] and is being explored as a potential treatment for EBVaGC. Although well-designed clinical trials confirming its efficacy in EBVaGC are lacking [38,39], transcriptional signatures, such as interleukin (IL)-12-mediated signaling and increased immune cell infiltration, provide a biological rationale for its use [2]. Preliminary clinical evidence also suggests promising outcomes for immunotherapy in EBVaGC [40,41]. Theoretically, EBV infection may be associated with PD-L1 expression, with both contributing to favorable immunotherapy responsiveness. However, this association remains controversial. While some studies have reported significant associations [5,7,8,9], other studies, including our study, did not [10,27]. Variability in PD-L1 testing methods, including clone selection, scoring systems, and tissue sampling (considering tumor heterogeneity), likely contribute to inconsistent outcomes across trials. Additionally, EBV-related immune activation may involve alternative pathways independent of PD-L1, such as RANBP2-mediated transcriptional repression, caspase activation, or IL-12-mediated signaling [2], which may still predict immunotherapy response despite PD-L1 negativity.

PD-L1 expression rates exhibit significant regional variability [18]. In our study, PD-L1 positivity using the 22C3 assay was 9.1% and 6.8% at CPS cutoffs of ≥1 and ≥5, respectively, which are markedly lower than the 14–69% range reported in other populations [18]. These discrepancies may reflect differences in ethnicity, clinical characteristics, tissue sampling methods, staining techniques, and scoring criteria. Interchangeability between the PD-L1 22C3 and 28-8 assays has been shown in lung cancer studies [42] and may be extrapolated to gastric cancer [43]. Only one prior study from Thailand reported PD-L1 positivity rates of 22% and 7% at CPS cutoffs of ≥1 and ≥5, respectively [44]. Although higher than our findings, these rates remain lower than those reported internationally, indicating potential geographic and ethnic influences on PD-L1 expression.

The prognostic role of PD-L1 expression in gastric cancer remains controversial. A meta-analysis associated PD-L1 positivity with poor prognosis but highlighted substantial heterogeneity (I² = 79%) among the included studies [18], with 11 studies linking PD-L1 overexpression to poor prognosis, 3 suggesting a favorable outcome, and 1 reporting no association with survival. In our study, no statistically significant association between PD-L1 expression and survival was observed, although a trend toward longer median OS in PD-L1-positive patients was noted. However, these findings should be interpreted with caution owing to the limited sample size and statistical power. Further studies are needed to clarify the prognostic significance of PD-L1 expression in gastric cancer while accounting for potential regional and methodological variations.

Although neither EBV nor PD-L1 expression was identified as a prognostic factor for OS in gastric cancer, localized-stage disease and good ECOG PS emerged as strong favorable prognostic indicators compared with metastatic-stage disease and poor ECOG PS. Additionally, our findings support hypoalbuminemia as a poor prognostic factor, consistent with the previous studies [45,46,47]. Our study also indicates the potential benefits of palliative gastrectomy in patients with metastatic-stage gastric cancer, aligning with the findings from prior studies [48,49]. This survival benefit may be attributed to the prevention of tumor-related complications, including bleeding-induced anemia, gastric outlet obstruction, causing severe malnutrition and cachexia, and intractable abdominal pain that unavoidably impairs quality of life. Surgical resection may also mitigate local complications and invasion, thereby improving survival. However, decisions regarding palliative gastrectomy should be individualized based on clinical status, comorbidities, and the availability of treatment options. More importantly, a multidisciplinary approach is crucial to balance potential benefits against risks, including surgical complications and delays in initiating systemic therapy.

Among patients with metastatic-stage gastric adenocarcinoma, those who received palliative chemotherapy had a median OS of 10.64 months, compared with 1.31 months for those receiving the best supportive care. These findings align with prior studies reporting a median OS of 10–12 months following palliative chemotherapy [50,51]. Current first-line treatment for metastatic gastric cancer includes targeted therapy, particularly for HER2-positive cases, and immunotherapy for PD-L1-positive disease, both of which have been shown to improve median OS to 13–14 months [15,16,17,52]. However, our study highlighted significant disparities in access to these therapies in Thailand. Only 20% of patients with metastatic gastric cancer in our study were tested for HER2 overexpression due to limited reimbursement for anti-HER2 therapy, which is restricted to the Civil Servant Medical Benefit Scheme. Most patients were covered by the universal healthcare scheme, which does not reimburse anti-HER2 therapy. Similarly, immunotherapy is not reimbursed under any circumstances in Thailand. Consequently, no patient in our cohort with PD-L1-positive metastatic gastric cancer received immunotherapy, although two patients with PD-L1-negative metastatic gastric cancer accessed such treatment through clinical trials. These findings underscore the challenges of biomarker testing and therapeutic access in resource-limited settings, emphasizing the need for policy changes to ensure equitable access to therapies for metastatic gastric cancer.

This study emphasizes the importance of biomarkers as a current and future cornerstone of personalized gastric cancer treatment, in line with the latest TCGA classification. PD-L1 is a validated predictive biomarker for immunotherapy, while EBV represents a novel molecular subtype with theoretical immunotherapeutic responsiveness. Notably, biomarker prevalence exhibits geographical variations. Our findings provide updated data on EBV and PD-L1 expression in patients with both localized- and metastatic-stage gastric cancer in Thailand, along with real-world survival outcomes. However, this single-center study is constrained by a small sample size, and the low prevalence of EBVaGC and PD-L1 positivity compared with previous reports may limit conclusions regarding their prognostic and predictive roles. Lastly, the limited use of immunotherapy in this cohort precluded the assessment of its potential benefits in the EBVaGC and PD-L1-positive subgroups.

## 5. Conclusions

In Thailand, EBVaGC and PD-L1-positive gastric cancer were observed in 4.5% and 9.1% of cases, respectively. No significant association was found between EBV status and PD-L1 expression, although both may confer favorable responses to immunotherapy. Therefore, EBV testing may serve as an additional biomarker for patient stratification in gastric cancer, independent of PD-L1 expression. Larger cohort studies, particularly those including patients receiving immunotherapy, are warranted to validate its prognostic and predictive value, potentially establishing the utility of EBV testing in clinical practice.

## Figures and Tables

**Table 1:** Baseline characteristics of patients.

	Localized Stage (*n* = 56)	Metastatic Stage (*n* = 72)	Total # (*n* = 132)
Median age, years (SD) *	65.4 (11.5)	60.9 (12.6)	63.2 (12.3)
Age ≥ 65 years, *n* (%)	27 (48.2)	30 (41.7)	60 (45.5)
Sex, *n* (%)			
Male	35 (62.5)	46 (63.9)	82 (62.1)
Female	21 (37.5)	26 (36.1)	50 (37.9)
ECOG PS, *n* (%) *			
0–1	45 (80.4)	33 (45.8)	79 (59.8)
≥2	11 (19.6)	39 (54.2)	53 (40.2)
BMI, n (kg/m^2^; %)			
<18.5	11 (19.6)	30 (41.7)	41 (31.3)
18.5–22.9	22 (39.3)	23 (31.9)	47 (35.9)
23.0–24.9	13 (23.2)	10 (13.9)	24 (18.3)
≥25	10 (17.9)	9 (12.5)	19 (14.5)
Smoking, *n* (%)			
Current or former	29 (51.8)	34 (47.2)	63 (47.7)
Never	26 (46.4)	34 (47.2)	63 (47.7)
Unknown	1 (1.8)	4 (5.6)	6 (4.6)
Alcohol drinking, *n* (%)			
Current or former	25 (44.6)	29 (40.3)	54 (40.9)
Never	30 (53.6)	39 (54.2)	72 (54.5)
Unknown	1 (1.8)	4 (5.6)	6 (4.6)
Laboratory at cancer diagnosis			
Hemoglobin, g/dL (IQR)	11.1 (9.6, 12.7)	10.6 (9.2, 12.0)	11.0 (9.3, 12.2)
Creatinine, mg/dL (IQR)	0.9 (0.7, 1.0)	0.8 (0.7, 1.0)	0.8 (0.7, 1.0)
CrCl < 60 mL/min, *n* (%)	21 (37.5)	26 (37.1)	49 (38.0)
Total bilirubin, mg/dL (IQR)	0.4 (0.3, 0.7)	0.4 (0.2, 0.7)	0.4 (0.3, 0.7)
Albumin, g/dL (SD) *	3.8 (0.6)	3.4 (0.6)	3.6 (0.7)
Tumor location, *n* (%)			
Proximal stomach	23 (41.1)	22 (30.6)	45 (34.1)
Middle stomach	18 (32.1)	17 (23.6)	36 (27.3)
Distal stomach	13 (23.2)	25 (34.7)	41 (31.1)
Diffuse/linitis plastica	2 (3.6)	8 (11.1)	10 (7.5)
T stage, *n* (%) *			
T1	3 (5.4)	0 (0)	3 (2.3)
T2	5 (8.9)	1 (1.4)	6 (4.5)
T3	21 (37.5)	13 (18.1)	34 (25.8)
T4	24 (42.9)	15 (20.8)	39 (29.5)
Tx	3 (5.4)	43 (59.7)	50 (37.9)
N stage, *n* (%) *			
N0	17 (30.4)	1 (1.4)	18 (13.6)
N1	15 (26.8)	14 (19.4)	29 (22.0)
N2	13 (23.2)	17 (23.6)	30 (22.7)
N3	11 (19.6)	11 (15.3)	22 (16.7)
Nx	0 (0)	29 (40.3)	33 (25.0)
Tumor differentiation, *n* (%)			
Well	8 (14.3)	6 (8.3)	16 (12.1)
Moderate	17 (30.4)	15 (20.8)	33 (25.0)
Poor	31 (55.4)	51 (70.8)	83 (62.9)
Signet ring cell feature, *n* (%)	13 (23.2)	20 (27.8)	34 (25.8)
Lauren classification, *n* (%) *			
Diffuse	20 (35.7)	47 (65.3)	69 (52.3)
Intestinal	26 (46.4)	14 (19.4)	41 (31.1)
Mixed	7 (12.5)	11 (15.3)	19 (14.1)
Unknown	3 (5.4)	(0)	3 (2.3)
Lymphovascular invasion, *n* (%) *			
Yes	29 (51.8)	12 (16.7)	41 (31.1)
No	18 (32.1)	0 (0)	18 (13.6)
Unknown	9 (16.1)	60 (83.3)	73 (55.3)
Perineural invasion, *n* (%) *			
Yes	22 (39.3)	9 (12.5)	31 (23.5)
No	25 (44.6)	3 (4.2)	28 (21.2)
Unknown	9 (16.1)	60 (83.3)	73 (55.3)
*Helicobacter pylori* infection, *n* (%)			
Yes	10 (17.9)	8 (11.1)	19 (14.4)
No	22 (39.3)	20 (27.8)	43 (32.6)
Unknown	24 (42.9)	44 (61.1)	70 (53.0)
HER2 overexpression, *n* (%) *			
Yes	1 (1.8)	0 (0)	1 (0.8)
No	4 (7.1)	21 (29.2)	25 (18.9)
Unknown	51 (91.1)	51 (70.8)	106 (80.3)

# Four patients classified as Mx were included in the total patient cohort. * Statistical significance between the localized-stage and metastatic-stage cohorts (*p* < 0.05). ECOG, Eastern Cooperative Oncology Group; PS, performance status; BMI, body mass index; CrCl, creatinine clearance; SD, standard deviation; IQR, interquartile range.

**Table 2:** Clinicopathological features related to EBV and PD-L1 status.

	EBV Status			PD-L1 Status		
	Positive (*n* = 6)	Negative (*n* = 126)	*p*-Value	Positive (CPS ≥ 1) (*n* = 12)	Negative (CPS < 1) (*n* = 120)	*p*-Value
Median age, years (SD)	70.6 (10.7)	62.8 (12.3)	0.133	67.7 (10.9)	62.7 (12.4)	0.186
Age ≥ 65 years, *n* (%)	4 (66.7)	56 (44.4)	0.410	7 (58.3)	53 (44.2)	0.525
Sex, *n* (%)			0.082			1.000
Male	6 (100)	76 (60.3)		8 (66.7)	74 (61.7)	
Female	0 (0)	50 (39.7)		4 (33.3)	46 (38.2)	
Smoking, *n* (%)			0.058			0.730
Current or former	6 (100)	57 (45.2)		6 (50.0)	57 (47.5)	
Never	0 (0)	63 (50.0)		6 (50.0)	57 (47.5)	
Unknown	0 (0)	6 (4.8)		0 (0)	6 (5.0)	
Tumor Location, *n* (%)			0.152			0.512
Proximal	2 (33.3)	43 (34.1)		3 (25.0)	42 (35.0)	
Middle	3 (50.0)	33 (26.2)		2 (16.7)	34 (28.3)	
Distal	1 (16.7)	40 (31.8)		5 (41.6)	36 (30.0)	
Diffuse	0 (0)	10 (7.9)		2 (16.7)	8 (66.7)	
T stage, *n* (%)			0.370			0.741
T1	0 (0)	3 (2.4)		0 (0)	3 (2.5)	
T2	0 (0)	6 (4.7)		0 (0)	6 (5.0)	
T3	3 (50.0)	31 (24.6)		4 (33.3)	30 (25.0)	
T4	0 (0)	39 (31.0)		2 (16.7)	37 (30.8)	
Tx	3 (50.0)	47 (37.3)		6 (50.0)	44 (36.7)	
N stage, *n* (%)			0.561			0.428
N0	1 (16.7)	17 (13.5)		2 (16.7)	16 (13.3)	
N1	0 (0)	29 (23.0)		2 (16.7)	27 (22.5)	
N2	1 (16.7)	29 (23.0)		5 (41.7)	25 (20.8)	
N3	1 (16.7)	21 (16.7)		2 (16.7)	20 (16.7)	
Nx	3 (50.0)	30 (23.8)		1 (8.3)	32 (26.7)	
M stage, *n* (%)			0.747			0.091
M0	2 (33.3)	55 (43.6)		7 (58.3)	49 (40.8)	
M1	4 (66.7)	68 (54.0)		5 (41.7)	67 (55.9)	
Mx	0 (0)	3 (2.4)		0 (0)	4 (3.3)	
Tumor differentiation, *n* (%)			0.704			0.822
Well	0 (0)	16 (12.7)		1 (8.3)	15 (12.5)	
Moderate	1 (16.7)	32 (25.4)		4 (33.3)	29 (24.2)	
Poor	5 (83.3)	78 (61.9)		7 (58.3)	76 (63.3)	
Signet ring cell feature, *n* (%)	0 (0)	34 (27.0)	0.338	4 (33.3)	30 (25.0)	0.505
Lauren classification, *n* (%)			0.261			0.617
Diffuse	4 (66.7)	65 (51.6)		5 (41.7)	64 (53.3)	
Intestinal	0 (0)	41 (32.5)		4 (33.3)	37 (30.8)	
Mixed	2 (33.3)	17 (13.5)		3 (25.0)	16 (13.4)	
Unknown	0 (0)	3 (2.4)		0 (0)	3 (2.5)	
Lymphovascular invasion, *n* (%)			0.860			0.483
Yes	2 (33.3)	39 (30.9)		2 (16.7)	39 (32.5)	
No	0 (0)	18 (14.3)		1 (8.3)	17 (14.2)	
Unknown	4 (66.7)	69 (54.8)		9 (75.0)	64 (53.3)	
Perineural invasion, *n* (%)			1.000			0.393
Yes	1 (16.7)	30 (23.8)		2 (16.7)	29 (24.2)	
No	1 (16.7)	27 (21.4)		1 (8.3)	27 (22.5)	
Unknown	4 (66.7)	69 (54.8)		9 (75.0)	64 (53.3)	
Helicobacter pylori infection, *n* (%)			1.000			0.070
Yes	1 (16.7)	18 (14.3)		3 (25.0)	16 (13.3)	
No	2 (33.3)	41 (32.5)		6 (50.0)	37 (30.9)	
Unknown	3 (50.0)	67 (53.2)		3 (25.0)	67 (55.8)	
HER2 overexpression, *n* (%)			0.613			0.073
Yes	0 (0)	1 (0.8)		1 (8.3)	0 (0)	
No	0 (0)	25 (19.8)		1 (8.3)	24 (20.0)	
Unknown	6 (100)	100 (79.4)		10 (83.4)	96 (80.0)	

EPV, Epstein–Barr virus; PD-L1, programmed death-ligand 1.

**Table 3:** Association between EBV status and PD-L1 expression in gastric cancer.

All Patient Cohort			
PD-L1 Expression	EBV Positive (*n* = 6)	EBV Negative (*n* = 126)	*p*-Value
CPS < 1	4 (66.7)	116 (92.1)	0.093
CPS ≥ 1	2 (33.3)	10 (7.9)	
CPS < 5	4 (66.7)	119 (94.4)	0.054
CPS ≥ 5	2 (33.3)	7 (5.6)	
CPS < 10	6 (100)	119 (94.4)	1.0
CPS ≥ 10	0 (0)	7 (5.6)	
Localized-stage cohort			
PD-L1 expression	EBV positive (*n* = 2)	EBV negative (*n* = 54)	*p*-value
CPS < 1	1 (50.0)	48 (88.9)	0.236
CPS ≥ 1	1 (50.0)	6 (11.1)	
CPS < 5	1 (50.0)	48 (88.9)	0.236
CPS ≥ 5	1 (50.0)	6 (11.1)	
CPS < 10	2 (100)	48 (88.9)	1.0
CPS ≥ 10	0 (0)	6 (11.1)	
Metastatic-stage cohort			
PD-L1 expression	EBV positive (*n* = 4)	EBV negative (*n* = 68)	*p*-value
CPS < 1	3 (75.0)	64 (94.1)	0.255
CPS ≥ 1	1 (25.0)	4 (5.9)	
CPS < 5	3 (75.0)	67 (98.5)	0.109
CPS ≥ 5	1 (25.0)	1 (1.5)	
CPS < 10	4 (100)	67 (98.5)	1.0
CPS ≥ 10	0 (0)	1 (1.5)	

EPV, Epstein–Barr virus; PD-L1, programmed death-ligand 1; CPS, combined positive score.

**Table 4:** Prognostic factors for overall survival.

Variables	Univariate		Multivariate	
	HR (95% CI)	*p*-Value	HR (95% CI)	*p*-Value
Age ≥ 65 years	1.18 (0.80, 1.74)	0.410		
BMI (kg/m^2^)				
<18.5	Ref			
18.5–22.9	0.64 (0.40, 1.02)	0.059		
23.0–24.9	0.82 (0.44, 1.54)	0.544		
≥25.0	0.60 (0.32, 1.13)	0.113		
ECOG PS		<0.001		<0.001
0–1	0.30 (0.20, 0.46)		0.37 (0.23, 0.61)	
≥2	Ref		Ref	
Lauren classification				
Diffuse	Ref			
Intestinal	0.66 (0.41, 1.06)	0.086		
Mixed	0.92 (0.53, 1.60)	0.769		
Tumor location				
Proximal	Ref		Ref	
Middle	1.31 (0.77, 2.24)	0.315	0.87 (0.46, 1.63)	0.665
Distal	1.15 (0.72, 1.84)	0.549	0.92 (0.54, 1.57)	0.758
Diffuse	2.23 (1.09, 4.57)	0.029	1.32 (0.60, 2.92)	0.486
Differentiation				
Well	Ref			
Moderate	0.85 (0.42, 1.70)	0.638		
Poor	0.96 (0.53, 1.74)	0.896		
Signet ring cell feature	0.82 (0.52, 1.29)	0.393		
Stage		<0.001		0.007
Localized stage	0.31 (0.18, 0.50)		0.22 (0.07, 0.66)	
Metastatic stage	Ref		Ref	
EBV positive	1.48 (0.60, 3.66)	0.400	0.78 (0.28, 2.19)	0.643
PD-L1-positive (CPS ≥ 1)	1.02 (0.49, 2.11)	0.953	0.99 (0.43, 2.29)	0.981
Hemoglobin < 10 g/dL	1.07 (0.70, 1.62)	0.758		
Albumin < 3.5 g/dL	1.78 (1.19, 2.67)	0.005	1.71 (1.05, 2.79)	0.032
Gastrectomy				
No	Ref		Ref	
Subtotal	0.32 (0.17, 0.59)	<0.001	1.07 (0.32, 3.59)	0.909
Total	0.33 (0.17, 0.65)	0.001	1.8 (0.52, 6.19)	0.354
Palliative	0.53 (0.28, 1.00)	0.051	0.51 (0.25, 1.04)	0.064

BMI, body mass index; ECOG, Eastern Cooperative Oncology Group; PS, performance status; EPV, Epstein–Barr virus; PD-L1, programmed death-ligand 1; CPS, combined positive score; HR, hazard ratio; CI, confidence interval.

## Data Availability

The datasets used and/or analyzed during the current study are available from the corresponding author upon reasonable request.

## Supplementary Materials

The following supporting information can be downloaded at: https://www.mdpi.com/article/10.3390/cancers17091492/s1, Figure S1: Median overall survival (OS) between patients with Epstein–Barr virus-associated gastric cancer (EBVaGC) and non-EBVaGC in localized and metastatic stages; Figure S2: Median OS between patients with PD-L1-positive (CPS ≥ 1) and negative (CPS < 1) gastric cancer in localized and metastatic stages; Figure S3: Median OS between patients who received palliative chemotherapy and those who received best supportive care in metastatic-stage gastric cancer; Figure S4: Median OS between patients with and without palliative gastrectomy in metastatic-stage gastric cancer; Figure S5: Median OS between patients who underwent gastrectomy with and without adjuvant chemotherapy and those who received palliative chemotherapy without gastrectomy in localized-stage gastric cancer; Table S1: Number and organ metastasis in metastatic-stage gastric cancer; Table S2: Treatment approaches for localized-stage gastric cancer; Table S3: Treatment approaches for metastatic-stage gastric cancer.

## Abbreviations

The following abbreviations are used in this manuscript:

BMI	Body mass index
CPS	Combined positive score
EBER	Epstein–Barr virus-encoded small RNA
EBV	Epstein–Barr virus
EBVaGC	Epstein–Barr virus-associated gastric cancer
ECOG	Eastern Cooperative Oncology Group
FFPE	Formalin-fixed paraffin-embedded tissue
IHC	Immunohistochemical
ISH	In situ hybridization
MSI	Microsatellite instability
PD-L1	Programmed death-ligand 1
TCGA	The Cancer Genome Atlas
