# Supplementary material for: Association Between Epstein–Barr Virus Infection and PD-L1 Expression in Gastric Cancer: Prevalence, Clinicopathological Features, and Prognostic Implications
Source: Cancers (Basel). 2025 Apr 29;17(9):1492. doi: 10.3390/cancers17091492 (PMC12070931; doi:10.3390/cancers17091492)
Supplement: Supplementary file 1 [file cancers-17-01492-s001.zip › cancers-3544860-supplementary.pdf]

Supplementary Material

Table S1. Number and organ metastasis in metastatic-stage gastric cancer

	**EBV status**		**PD-L1 status**		**Total (n = 72)**
	**Positive (n = 4)**	**Negative (n = 68)**	**Positive (CPS ≥ 1) (n = 5)**	**Negative (CPS < 1) (n = 67)**	
Number of organ metastasis, n (%)					
1	2 (50.0)	43 (63.2)	1 (20.0)	44 (65.7)	45 (62.5)
2	1 (25.0)	15 (22.1)	2 (40.0)	14 (20.9)	16 (22.2)
≥3	1 (25.0)	10 (14.8)	2 (40.0)	9 (6.0)	11 (15.3)
Organ metastasis, n (%)					
Lung	3 (75.0)	11 (16.2)	1 (20.0)	13 (19.4)	14 (19.4)
Lymph node	0 (0)	23 (33.8)	1 (20.0)	22 (32.8)	33 (31.9)
Liver	2 (50.0)	15 (22.1)	3 (60.0)	14 (20.9)	17 (23.6)
Peritoneum	1 (25.0)	42 (61.8)	3 (60.0)	40 (59.7)	43 (59.7)
Pleura	1 (25.0)	4 (5.9)	1 (20.0)	4 (6.0)	5 (6.9)
Bone	0 (0)	6 (8.8)	2 (40.0)	4 (6.0)	6 (8.3)
Adrenal	0 (0)	3 (4.4)	0 (0)	3 (4.5)	3 (4.2)
Ovary	0 (0)	3 (4.4)	0 (0)	3 (4.5)	3 (4.2)

EBV, Epstein–Barr virus; CPS, combined positive score; PD-LI, programmed death-ligand 1

Table S2. Treatment approaches for localized-stage gastric cancer

	EBV status		PD-L1 status		Total (n = 56)
	Positive (n = 2)	Negative (n = 54)	Positive (CPS ≥ 1) (n = 7)	Negative (CPS < 1) (n = 49)	
**Surgery, n (%)**					
Total gastrectomy	2 (100)	18 (33.3)	1 (14.3)	19 (38.8)	20 (35.7)
Subtotal gastrectomy	0 (0)	29 (53.7)	2 (28.6)	27 (55.1)	29 (51.8)
No surgery	0 (0)	6 (11.1)	3 (42.9)	3 (6.1)	6 (10.7)
Unknown	0 (0)	1 (1.9)	1 (14.3)	0 (0)	1 (1.8)
**LN dissection, n (%)**	Total = 2	Total = 47	Total = 3	Total = 46	Total = 49
D2 resection	2 (100)	29 (61.7)	3 (100)	28 (60.9)	31 (63.3)
D1 resection	0 (0)	18 (33.3)	0 (0)	18 (39.1)	18 (36.7)
**Systemic therapy, n (%)**					
Adjuvant chemotherapy	2 (100)	22 (40.7)	3 (42.9)	21 (42.9)	24 (42.9)
Neoadjuvant chemotherapy	0 (0)	10 (18.5)	1 (14.3)	9 (18.4)	10 (17.9)
Definitive CCRT	0 (0)	1 (1.9)	0 (0)	1 (2.0)	1 (1.8)
Palliative chemotherapy	0 (0)	5 (9.3)	3 (42.9)	2 (4.1)	5 (8.9)
No chemotherapy	0 (0)	14 (25.9)	0 (0)	14 (28.6)	14 (25.0)
Unknown	0 (0)	2 (3.7)	0 (0)	2 (4.1)	2 (3.6)
**Chemotherapy regimen, n (%)**	Total = 2	Total = 38	Total = 7	Total = 33	Total = 40
FOLFOX	0 (0)	7 (18.4)	1 (14.3)	6 (18.2)	7 (17.5)
CAPOX	1 (50.0)	8 (21.1)	3 (42.9)	6 (18.2)	9 (22.5)
Cisplatin plus 5-FU	0 (0)	7 (18.4)	0 (0)	7 (21.1)	7 (17.5)
Carboplatin plus 5-FU	0 (0)	3 (7.9)	0 (0)	3 (9.1)	3 (7.5)
5-FU	0 (0)	4 (10.5)	0 (0)	4 (12.1)	4 (10.0)
FLOT	0 (0)	2 (5.3)	1 (14.3)	1 (3.0)	2 (5.0)
TS-1	1 (50.0)	5 (13.2)	1 (14.3)	5 (15.2)	6 (15.0)
Gemcitabine	0 (0)	1 (2.6)	0 (0)	1 (3.0)	1 (2.5)
Carboplatin plus paclitaxel	0 (0)	1 (2.6)	1 (14.3)	0 (0)	1 (2.5)
**Discontinuation, n (%)**	Total = 2	Total = 38	Total = 7	Total = 33	Total = 40
Complete treatment	1 (50.0)	23 (60.5)	1 (14.3)	23 (69.7)	24 (60.0)
Progressive disease	1 (50.0)	3 (7.9)	1 (14.3)	3 (9.1)	4 (10.0)
Death	0 (0)	1 (2.6)	1 (14.3)	0 (0)	1 (2.5)
Worsening ECOG PS	0 (0)	5 (13.2)	2 (28.6)	3 (9.1)	5 (12.5)
Patient preference	0 (0)	2 (5.3)	1 (14.3)	1 (3.0)	2 (5.0)
Loss of follow-up	0 (0)	2 (5.3)	0 (0)	2 (6.1)	2 (5.0)
Refer to another hospital	0 (0)	2 (5.3)	1 (14.3)	1 (3.0)	2 (5.0)
**Recurrent disease, n (%)**					
Yes	1 (50.0)	20 (37.0)	1 (14.3)	20 (40.8)	21 (37.5)
No	1 (50.0)	28 (51.9)	3 (42.9)	26 (53.1)	29 (51.8)
Unknown	-	6 (11.1)	3 (42.9)	3 (6.1)	6 (10.7)
**First-line systemic treatment for recurrent metastatic disease, n (%)**	Total = 1	Total = 20	Total = 1	Total = 20	Total = 21
FOLFOX	-	2 (10.0)	-	2 (9.1)	2 (9.5)
Paclitaxel	-	2 (10.0)	-	2 (9.1)	2 (9.5)
Paclitaxel plus ramucirumab	-	1 (5.0)	-	1 (4.5)	1 (4.8)
No	-	5 (25.0)	-	5 (22.7)	5 (23.8)
Unknown	1 (100)	10 (50.0)	1 (100)	10 (45.5)	11 (52.4)

Table S3. Treatment approaches for metastatic-stage gastric cancer

	EBV status		PD-L1 status		Total (n = 72)
	Positive (n = 4)	Negative (n = 68)	Positive (CPS ≥ 1) (n = 5)	Negative (CPS < 1) (n = 67)	
**Surgery, n (%)**					
Palliative gastrectomy	0 (0)	11 (16.2)	0 (0)	11 (16.4)	11 (15.3)
No surgery	4 (100)	57 (83.8)	5 (100)	56 (83.6)	61 (84.7)
**Systemic therapy, n (%)**					
Palliative systemic therapy	1 (25.0)	32 (47.1)	3 (60.0)	30 (44.8)	33 (45.8)
Best supportive care	3 (75.0)	33 (48.5)	2 (40.0)	34 (50.7)	36 (50.0)
Unknown	0 (0)	3 (4.4)	0 (0)	3 (4.5)	3 (4.2)
**First-line chemotherapy, n (%)**	Total = 1	Total = 32	Total = 3	Total = 30	Total = 33
FOLFOX	0 (0)	14 (43.8)	1 (33.3)	13 (43.3)	14 (42.4)
CAPOX	0 (0)	7 (21.9)	0 (0)	7 (23.3)	7 (21.2)
SOX	0 (0)	1 (3.1)	0 (0)	1 (3.3)	1 (3.0)
Cisplatin plus 5-FU	0 (0)	2 (6.2)	0 (0)	2 (6.7)	2 (6.1)
Carboplatin plus 5-FU	1 (100)	7 (21.9)	2 (66.7)	6 (20.0)	8 (24.2)
ECF	0 (0)	1 (3.1)	0 (0)	1 (3.3)	1 (3.0)
**Combined targeted therapy, n (%)**	Total = 1	Total = 32	Total = 3	Total = 30	Total = 33
Bemarituzumab	0 (0)	2 (6.2)	0 (0)	2 (6.7)	2 (6.1)
Zolbituximab	0 (0)	3 (9.4)	0 (0)	3 (10.0)	3 (9.1)
**Combined immunotherapy, n (%)**	Total = 1	Total = 32	Total = 3	Total = 30	Total = 33
Nivolumab	0 (0)	2 (6.2)	0 (0)	2 (6.7)	2 (6.1)
**Discontinuation, n (%)**	Total = 1	Total = 32	Total = 3	Total = 30	Total = 33
Complete treatment	0 (0)	5 (15.6)	0 (0)	5 (16.7)	5 (15.2)
Progressive disease	1 (100)	11 (34.4)	2 (66.7)	10 (33.3)	12 (36.4)
Death	0 (0)	2 (6.2)	0 (0)	2 (6.7)	2 (6.1)
Worsening ECOG PS	0 (0)	9 (28.1)	0 (0)	9 (30.0)	9 (27.3)
Patient preference	0 (0)	2 (6.2)	1 (33.3)	1 (3.3)	2 (6.1)
Loss of follow-up	0 (0)	1 (3.1)	0 (0)	1 (3.3)	1 (3.0)
Refer to another hospital	0 (0)	2 (6.2)	0 (0)	2 (6.7)	2 (6.1)
**Second-line chemotherapy, n (%)**	Total = 1	Total = 32	Total = 3	Total = 30	Total = 33
Paclitaxel	1 (100)	7 (21.9)	2 (66.7)	6 (20.0)	8 (24.2)
Paclitaxel plus ramucirumab	0 (0)	1 (3.1)	0 (0)	1 (3.3)	1 (3.0)
**Third-line chemotherapy, n (%)**	Total = 1	Total = 32	Total = 3	Total = 30	Total = 33
FOLFIRI	0 (0)	2 (6.25)	0 (0)	2 (6.7)	2 (6.1)

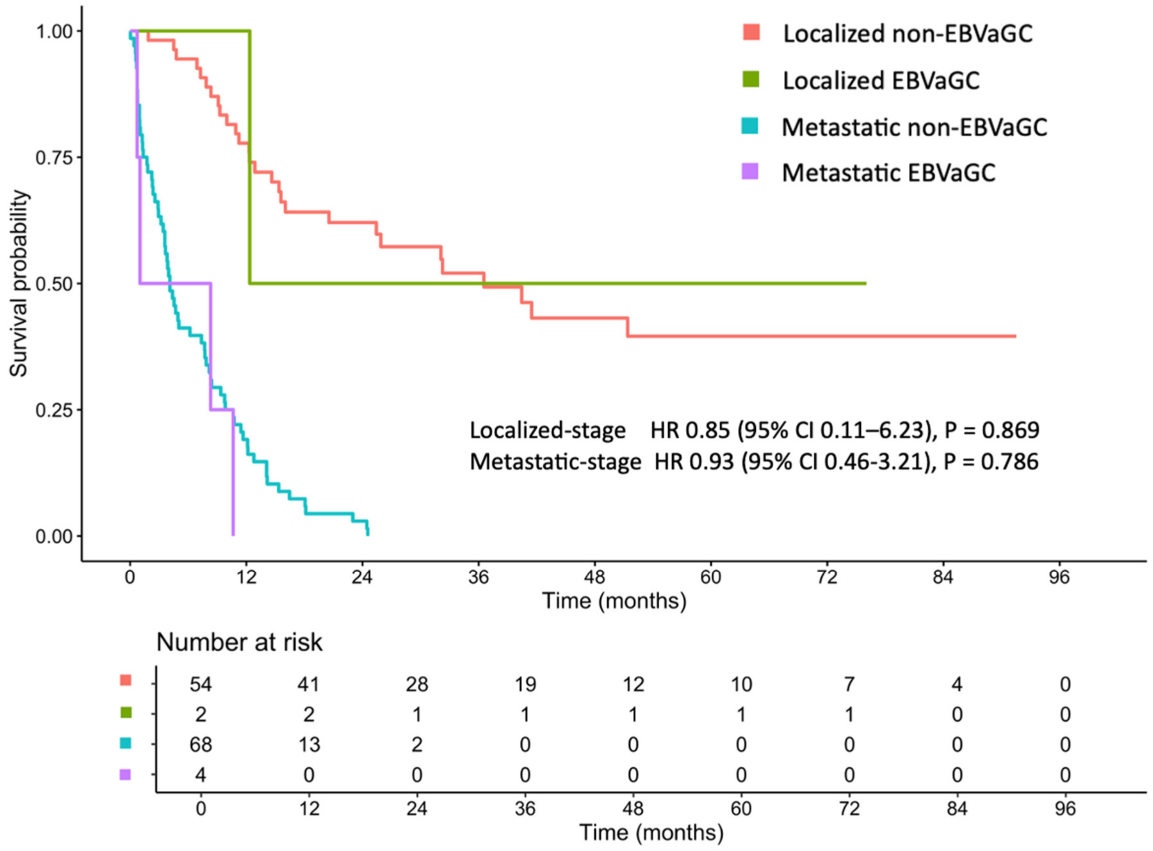

Figure S1. Median overall survival (OS) between patients with Epstein–Barr virus-associated gastric cancer (EBVaGC) and non-EBVaGC in localized and metastatic stages

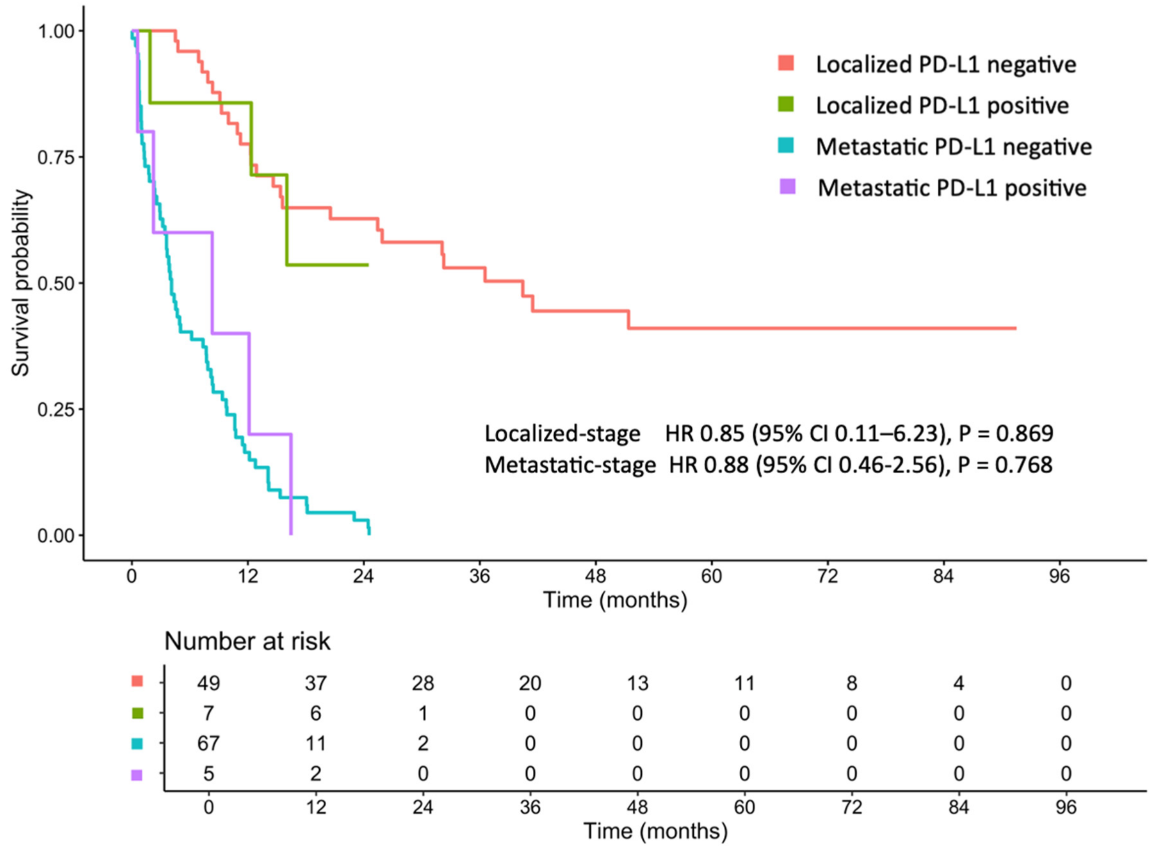

Figure S2. Median OS between patients with PD-L1 positive (CPS ≥ 1) and negative (CPS < 1) gastric cancer in localized and metastatic stages

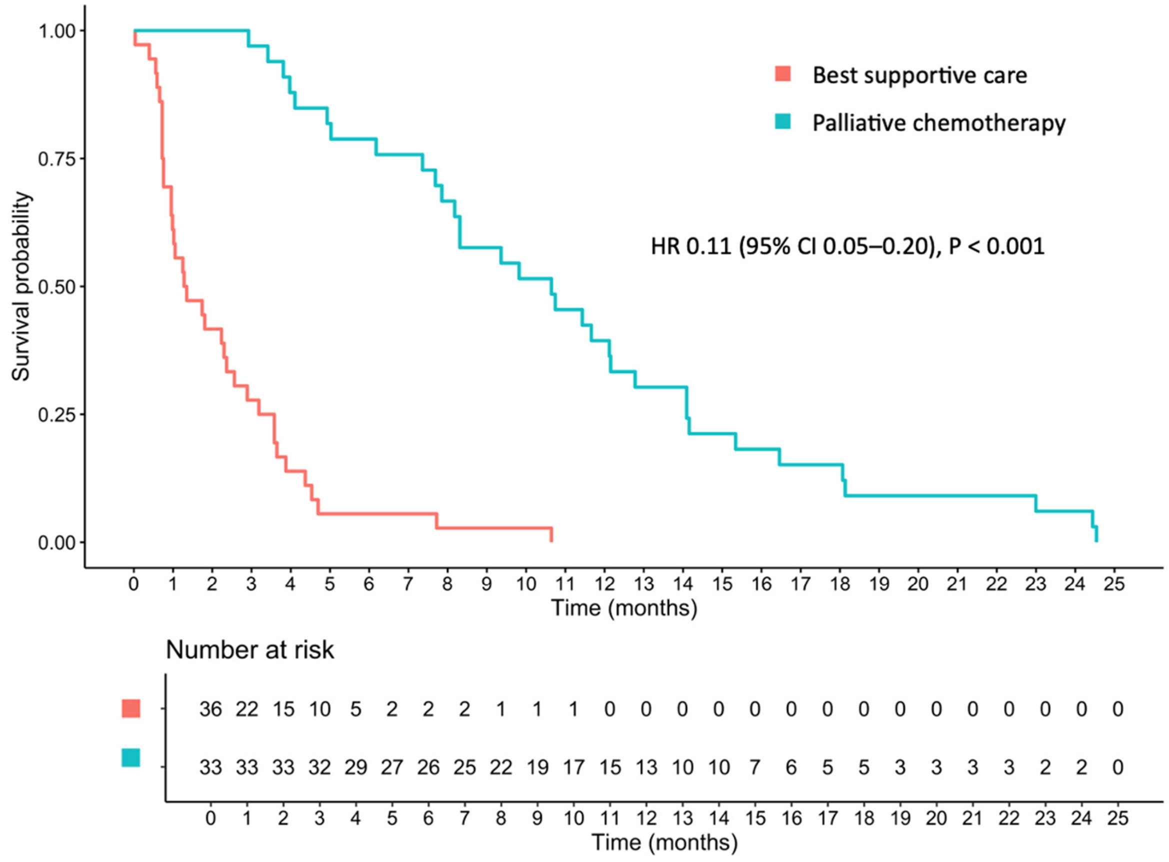

Figure S3. Median OS between patients who received palliative chemotherapy and those who received best supportive care in metastatic-stage gastric cancer

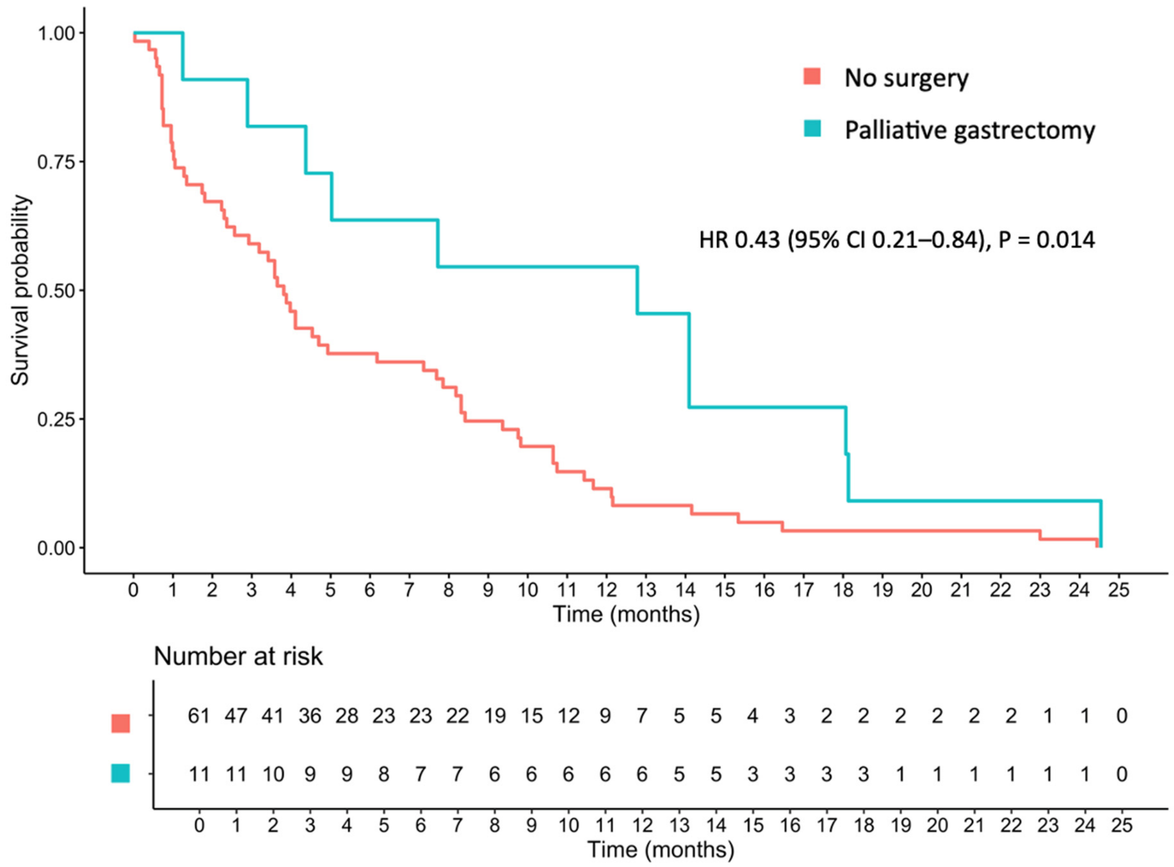

Figure S4. Median OS between patients with and without palliative gastrectomy in metastatic-stage gastric cancer

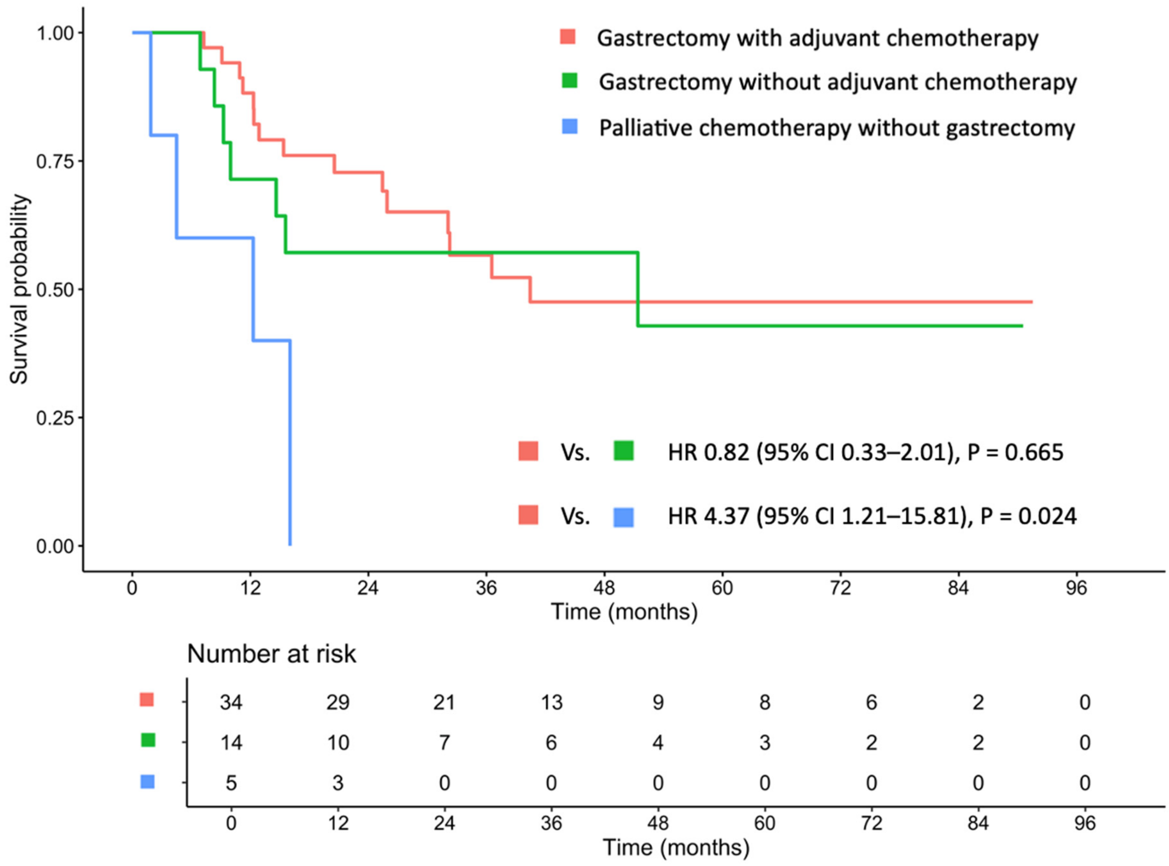

Figure S5. Median OS between patients who underwent gastrectomy with and without adjuvant chemotherapy and those who received palliative chemotherapy without gastrectomy in localized-stage gastric cancer
